# EspcTM: Kinetic Transition Network Based on Trajectory Mapping in Effective Energy Rescaling Space

**DOI:** 10.3389/fmolb.2020.589718

**Published:** 2020-10-27

**Authors:** Zhenyu Wang, Xin Zhou, Guanghong Zuo

**Affiliations:** ^1^T-Life Research Center, State Key Laboratory of Surface Physics, Department of Physics, Fudan University, Shanghai, China; ^2^School of Physical Sciences, University of Chinese Academy of Sciences, Beijing, China

**Keywords:** effective energy, molecular dynamics, trajectory mapping, Markov models, alanine dodecapeptide, transition network

## Abstract

The transition network provides a key to reveal the thermodynamic and kinetic properties of biomolecular systems. In this paper, we introduce a new method, named effective energy rescaling space trajectory mapping (EspcTM), to detect metastable states and construct transition networks based on the simulation trajectories of the complex biomolecular system. It mapped simulation trajectories into an orthogonal function space, whose bases were rescaled by effective energy, and clustered the interrelation between these trajectories to locate metastable states. By using the EspcTM method, we identified the metastable states and elucidated interstate transition kinetics of a Brownian particle and a dodecapeptide. It was found that the scaling parameters of effective energy also provided a clue to the dominating factors in dynamics. We believe that the EspcTM method is a useful tool for the studies of dynamics of the complex system and may provide new insight into the understanding of thermodynamics and kinetics of biomolecular systems.

## Introduction

The biomolecules are fundamentally dynamic in nature (Chodera et al., 2007). Protein folding, for example, involves the conformation change from polypeptide chain to a particular tertiary topology over microseconds to seconds, a process that can go awry and lead to misfolding and cause disease (Chiti and Dobson, 2006; Gregersen et al., 2006; Chodera et al., 2007; Guo et al., 2012; Wei et al., 2016; Zhou et al., 2019). Allosteric enzyme catalysis involves transitions between multiple conformational substates, only a few of which may allow substate access or catalysis (Eisenmesser et al., 2002; Boehr et al., 2006; Buch et al., 2011). Protein–ligand binding may alter the transition kinetics among multiple conformational states; for example, intrinsically disordered protein may have structured and unstructured binding pathways (Ithuralde et al., 2016; Paul et al., 2017; Li et al., 2019; Pan et al., 2019; Weng and Wang, 2020). Understanding of biomolecular dynamics is pivotal to reveal the function of biomolecules. Computer simulations of biomolecules, which made the biomolecular dynamics visible *in silico*, provide valuable insight for understanding how the dynamics of biomolecules drives biology processes (Cheatham and Kollman, 2000; Mirny and Shakhnovich, 2001; Norberg and Nilsson, 2002; Moraitakis et al., 2003; Levy et al., 2004; Zhou et al., 2004; Gao et al., 2005; Zuo et al., 2006, 2009; Li et al., 2008, 2013; Miyashita et al., 2009; Yang et al., 2014; Yan and Wang, 2019; Wu et al., 2020). In particular, molecular dynamics (MD) simulations can provide atomic-level details that are not always accessible in experiments and make this technique inevitable (Karplus and McCammon, 2002; Adcock and McCammon, 2006; Wang et al., 2009; Zuo et al., 2013). However, too many details will disguise the meaningful information. In most cases, the functional processes of biomolecules, the most interesting or important processes, correspond to slow dynamical processes. To extract these processes from numerous MD simulation trajectories, much effort has been involved in the development of methods for massive high-dimensional simulation data analysis. It was now well established from a variety of studies that an intelligible picture of the dynamics of biomolecules can be described as a transition network between several metastable states based on the simulation trajectories (Zwanzig, 1983; Kampen, 2007).

Markov state model (MSM) provides a powerful framework for analyzing dynamics of biosystems, such as MD simulations, to construct a transition network of metastable states. It has gained widespread use over the past several decades (Chodera et al., 2007; Gfeller et al., 2007; Noe et al., 2007; Bowman and Pande, 2010; Pande et al., 2010; Rao and Karplus, 2010; Bowman et al., 2013; Deng et al., 2013; Weber et al., 2013; Husic and Pande, 2018; Wang et al., 2018; Sengupta et al., 2019). In the analyzing process of MSM, the simulation conformations were first classified into thousands of small groups, named as microstates, by a geometric clustering method wherein these conformations were similar in geometry (Bowman et al., 2009; Pande et al., 2010). These microstates would be further clustered into several macrostates by standard spectral clustering method based on their transition frequency (Deuflhard and Weber, 2005; Chodera et al., 2007; Gfeller et al., 2007; Noe et al., 2007; Noe, 2008; Bowman and Pande, 2010; Pande et al., 2010; Rao and Karplus, 2010; Zuo et al., 2010; Bowman et al., 2013; Deng et al., 2013; Roblitz and Weber, 2013; Weber et al., 2013; Husic and Pande, 2018; Wang et al., 2018; Sengupta et al., 2019). Then, the transition network between the macrostates was reconstructed accordingly (Jayachandran et al., 2006; Buchete and Hummer, 2008; Prinz et al., 2011). Gong and Zhou (2010) presented the trajectory mapping (TM) method to construct a kinetic transition network of metastable states. Compared with MSM, TM grouped simulation trajectory pieces rather than individual conformations. They mapped the averaged conformation of each MD trajectory segment as a vector and calculate the principal components (PCs) of the trajectory-mapped vectors by the principal component analysis (PCA). The similar trajectory-mapped vectors were then grouped as metastable states by spectral clustering method, and transition events in simulation trajectories were further identified (Gong et al., 2015; Zhang et al., 2017; Zhang et al., 2019a; Zhang et al., 2019b).

In both MSM and TM methods, the discretization of MD trajectories, i.e., clustering of structures, plays a vital role in the analysis of MD trajectories. To make clustering of structures as accurate as possible, a variety of structural metrics and their functions were employed in analysis, for example, the torsion angles of backbone, the proportion of native contacts, root mean square deviation, and solvated energy (Gong et al., 2015). These analyses can be effective when all input coordinates are sufficient and irrelevant to each other. Thus, PCA was used to find orthogonal collective coordinates, which are linear combinations of the input coordinates and covered most of variances with only the first several eigenvectors (Lever et al., 2017). However, as mentioned above, the slow dynamical process is the concerned part in most cases. It is not always true that the high variance directions correspond to the kinetically slow-motion mode. Thus, some methods have been developed to obtain slow-motion directions. In the MSM, time-structure based Independent Correlation Analysis (tICA) was used (Naritomi and Fuchigami, 2011, 2013; Perez-Hernandez et al., 2013; Schwantes and Pande, 2013). It finds the slow collective coordinates by eigen-decomposition of a Δ*t*-interval autocorrelation matrix. In the TM, the averaged conformation of every τ-length MD trajectory segment was mapped as a vector in feature space to compose samples for the PCA method. It was argued that fast conformational fluctuations were suppressed after the segment averaging, and the PCs mainly involve slow motions (Zhang et al., 2017). In both tICA-MSM and TM methods, a hyper-parameter, Δ*t* for tICA-MSM and τ for TM, is required. It is difficult for inexperienced users. It is possible to obtain the optimized model by an automated process instead of a process of trial and error. For example, one might consider weighting the input coordination by an order parameter relevant to the functional processes of biomolecules, so that the input coordinates with high correlation contribute the most to the distance calculation and make the clustering effective and efficient to catch the functional processes, i.e., slow-motion patterns of the biomolecular system.

In this paper, we will present a new method, named effective energy rescaling space trajectory mapping (EspcTM), for detecting metastable states and constructing transition networks. It is a parameter-free analysis framework based on the previous TM method. In the EspcTM method, every snapshot of the trajectories was described by a high-dimensional vector and mapped into an orthogonal functional space. Different from the TM method, the features were rescaled by the effective energy of the dynamics to make the space effective to describe the slow processes of the system, and no hyperparameter was required. Here, the effective energy, which was filtered from the total potential energy of simulation trajectories by fast Fourier transform (FFT) and multiple linear regression, is an efficacious order parameter to describe the slow conformational change of complex system. The PCA method was also employed for dimensionality reduction and orthogonalization of the functional space. The metastable states were assigned by a spectral clustering method based on projections of the trajectories in this feature space. Then, the Markov transition matrix is constructed based on the transitions between these metastable states. We show application of this method by the movement of a Brownian particle and conformational dynamics of an alanine dodecapeptide (Ala_12_). It revealed their metastable states and kinetic transition network, as well as provided additional insight into the dynamics of these two systems.

## Theory and Method

**FIGURE 1:**
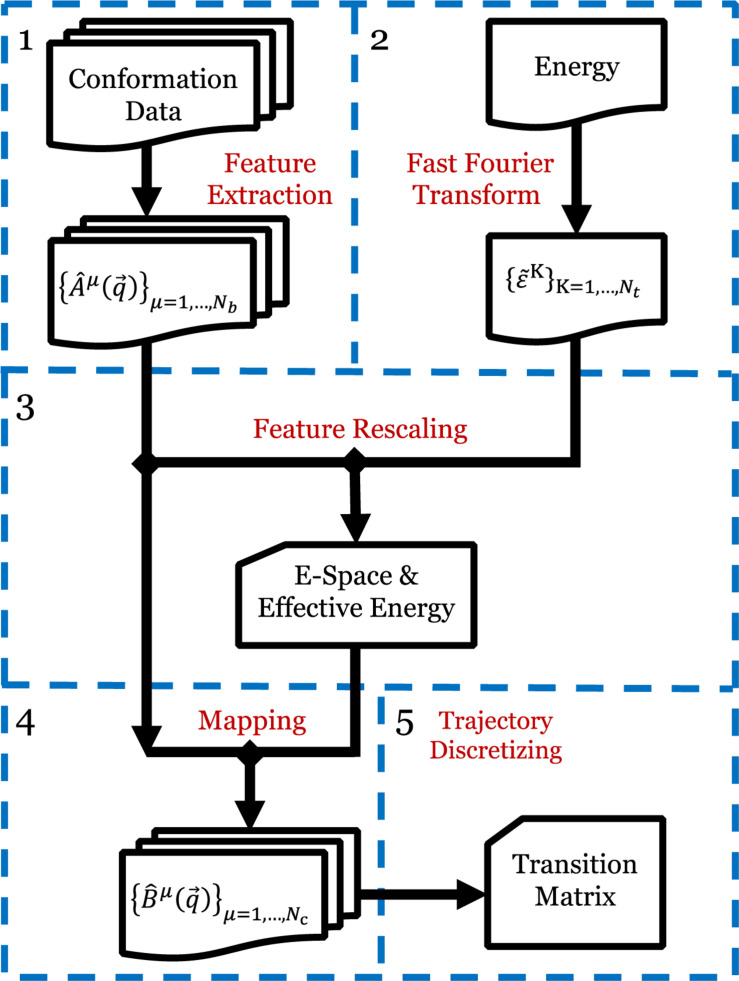
Flow chart of EspcTM method. Step 1: Extracting the conformational metrics with a set of basis functions for all simulation trajectories. Step 2: Extracting the potential energy to ${\left\{{\tilde{\varepsilon }}^{\text{K}}\right\}}_{K=1,\ldots ,N_{t}}$ by fast Fourier transform. Step 3: Multiple linear regression ${\tilde{\varepsilon }}^{\text{K}}$ and features, obtaining effective energy and E-space. Step 4: Mapping all trajectories to E-space. Step 5: Discretizing the trajectories based on the projections in E-space, and calculating the Markov transition matrix.

The EspcTM method is an analysis framework to identify metastable states from simulation data in the effective energy rescaling space and construct the transition network between the states based on the theory of Markov chain. In the EspcTM, an ordered parameter, named effective energy, was introduced to rescale feature space of the system. The simulation trajectories were mapped into the space and discretized to obtain the kinetic transition network of the system based on Markov chain theory. Figure 1 shows the flow chart of the EspcTM method, and details of the key steps are followed.

### Feature Extraction

In our study, there were *N*_*t*_ frames in every trajectory. They were mapped into a space consisting of *N*_*b*_ basis functions ${\left\{{\hat{A}}^{\mu }\,\left(\vec{q}\right)\right\}}_{\mu =1,\ldots ,N_{b}}$. To eliminate the effect of various units of basis function, normalization was performed on every dimension. Then, every trajectory was described as an *N*_*t*_×*N*_*b*_-dimension matrix in the feature space, i.e., feature matrix

(1) $$V=\left({\hat{A}}^{1}\,\left(\vec{q}\right),{\hat{A}}^{2}\,\left(\vec{q}\right),{\hat{A}}^{3}\,\left(\vec{q}\right),\ldots ,{\hat{A}}^{N_{b}}\,\left(\vec{q}\right)\right)$$

where $\vec{q}$ denotes the structural metrics, such as the torsion angle of backbone in peptide. Here, the basis functions ${\left\{{\hat{A}}^{\mu }\,\left(\vec{q}\right)\right\}}_{\mu =1,\ldots ,N_{b}}$ should be chosen to identify typical conformational motions of systems. In this work, we used the sine and cosine of structural metrics as the feature space (Gong and Zhou, 2010; Gong et al., 2015).

### Noise Reduction

It is obvious that every basis possesses different weight on describing the dynamics of complex system. It was argued that dynamics of complex systems, such as protein folding, can resemble a diffusive process on a rugged landscape of free energy (Onuchic et al., 1997). Thus, energy is an appropriate measure to rescale their coordinates. Most studies of complex system focus on the dynamics of a part of the system, and the rest of the system was regarded as the environment of the study object. For example, studies on protein folding focus on protein molecules. The conformational change of protein in protein folding is the interesting part, instead of the fluctuation of water molecules. However, the atoms of the system interacted with each other in a complicated way. The energy variation caused by the dynamics of the studied object is coupled with the energy caused by the fluctuation of the remaining part. It is difficult to isolate the meaningful energy in a frame without additional hypotheses. On the other hand, as mentioned above, the kinetic slowness is the main character of the interesting processes. Therefore, the dynamics of the important processes can be separated from the fluctuation in the frequency domain, where slow motion is treated as low-frequency signal and fluctuation can be filtered out as high-frequency noise.

In this work, FFT (Cochran et al., 1967) was applied to transform the energy of trajectories into frequency space. For every trajectory, the coefficients of frequencies were obtained by

(2) $${\tilde{\omega }}_{k}=\sum_{n=0}^{N_{t}-1}\varepsilon_{n}\cdot e^{-i\,n\,\omega_{k}}$$

Here, $i=\sqrt{-1}$ is the imaginary mark, *n* is the index of frames for the trajectory, *ε_n_* is the total potential energy of the *n*th frame obtained from the simulation data, *N*_*t*_ is the number of frames of a trajectory, and *ω*_*k*_ = 2*πk*/*N*_*t*_ corresponds to a frequency. To reduce the false edge, even extension was used before FFT for every trajectory. Then, a reverse FFT was performed on the first K frequencies for every trajectory to obtain the ${\tilde{\varepsilon }}^{\text{K}}$ of every frame:

(3) $${\tilde{\varepsilon }}_{n}^{\text{K}}=\sum_{k=0}^{\text{K}-1}{\tilde{\omega }}_{k}\cdot e^{i\,n\,\omega_{k}}$$

The fluctuation whose ω ≥*ω*_K_ was excluded in ${\tilde{\varepsilon }}^{\text{K}}$. To determine the number K, we performed multiple linear regression (Schneider et al., 2010) between K-energy vector ${\tilde{\varepsilon }}^{\text{K}}$ and feature matrix *V* for all trajectories:

(4) $${\tilde{\varepsilon }}^{\text{K}}=a_{0}^{\text{K}}+V\cdot {\hat{a}}^{\text{K}}+\epsilon^{\text{K}}$$

Here, $a_{0}^{\text{K}}$ (scalar) and ${\hat{a}}^{\text{K}}$ (*N*_*b*_-dimensional vector) are the fitting parameters, and *ϵ*^K^ is the error for the multiple linear regression. The effective energy $\tilde{\varepsilon }={\tilde{\varepsilon }}^{{\text{K}}^{*}}-\epsilon^{{\text{K}}^{*}}$ with the K^*^ = *arg*⁡*max*⁡*r*(*K*). Here, $r\,\left(K\right)=\sqrt{1-\bar{{\left(\epsilon^{\text{K}}\right)}^{2}}/{\left(\sigma^{\text{K}}\right)}^{2}}$ is the multiple correlation coefficient, (σ^K^)^2^ is the variance of ${\tilde{\varepsilon }}^{\text{K}}$, and *r=0* for the case ${\left(\sigma^{\text{K}}\right)}^{2}=\bar{{\left(\epsilon^{\text{K}}\right)}^{2}}=0$. For multiple trajectories, the FFT was performed on every trajectory separately. Due to same length and time interval of all trajectories in our study, all trajectories were mapped into the same frequency space {*ω*_*k*_}_*k* = 1,…,*Nt*_. Thus, in the revised FFT, the K-energies of all trajectories are the summary of the same frequencies for every K. Before multiple linear regression, K-energy vectors ${\tilde{\varepsilon }}^{\text{K}}$ and feature matrixes *V* of all trajectories were joined into a vector and a feature matrix for equation (4).

### Feature Rescaling and Mapping

The regression coefficients ${\hat{a}}^{\text{K}}$ were used as the weight factors on features. Every trajectory was described as a new *N*_*t*_×*N*_*b*_-dimension matrix:

(5) $$\tilde{V}=\text{V}\cdot d\,i\,a\,g\,\left({\hat{a}}^{\text{K}}\right)$$

Here, $d\,i\,a\,g\,\left({\hat{a}}^{\text{K}}\right)$ is an *N*_*b*_×*N*_*b*_ diagonal matrix with the elements of ${\hat{a}}^{\text{K}}$ on its main diagonal. A PCA (Sims et al., 2005) was applied to reduce the dimension and orthogonalize the components of all trajectories $\tilde{V}$. Descending according to eigenvalues, the first *N*_*c*_ eigenvectors were selected to consist of an *N*_*b*_×*N*_*c*_ matrix *M*. Here, *N*_*c*_≪*N*_*b*_, and *M* is the mapping operator, which reduced the *N*_*b*_−*dimension* vectors into *N*_*c*_−*dimension*, given top *N*_*c*_ eigenvalues whose sum has over 90% fraction of the sum of all eigenvalues. Here, we named this *N*_*c*_−*dimension* space as E-space since its input coordinates were weighted by the regression coefficients. By using the mapping operator *M*, we mapped all original feature matrixes *V*_*j*_ into the E-space. Therefore, every frame of the trajectories was described as an *N*_*c*_−*dimension* vector ${\left\{{\hat{B}}^{\mu }\,\left(\vec{q}\right)\right\}}_{\mu =1,\ldots ,N_{c}}$.

### Trajectory Discretizing

The clustering of conformations was performed in the E-space, i.e., based on the analysis of the projection vectors ${\left\{{\hat{B}}^{\mu }\,\left(\vec{q}\right)\right\}}_{\mu =1,\ldots ,N_{c}}$. Similar to the TM method (Gong and Zhou, 2010; Zhang et al., 2017), every trajectory was divided into a lot of isometric pieces, and the similarity between each two pieces was defined by their average vectors:

(6) S(t,t)′=∑i[Bi(t)Bi(t)′+1]∑i[Bi⁢(t)⁢Bi⁢(t)+1]×∑i[Bi(t)′Bi(t)′+1]

Here, we replaced the vectors of frames by the average vectors of trajectory pieces. It reduced the size of the similarity matrix and cost of computation resource. In practice, the length of the trajectory pieces can be varied in a reasonable range. The Robust Perron Cluster Analysis (PCCA+) method (Roblitz and Weber, 2013), implemented in pyEMMA (Scherer et al., 2015), was used to classify all pieces into *N*_*s*_ states based on the similarity matrix. Here, the number of states *N*_*s*_ was determined by the distribution of the eigenvalues of the similarity matrix (Roblitz and Weber, 2013). The Markov transition matrix *P* was obtained based on the discretized trajectories (Prinz et al., 2011). Since *P* is a row stochastic matrix, its largest left eigenvalue is 1. If there is a unique stationary distribution, it is true for our case, then the largest eigenvalue and the corresponding eigenvector is unique too. As the theory of stochastic process, the stationary distribution of the Markov process corresponds to the distribution of equilibrium state. More interestingly, the Markov transition matrix can also be used to reveal the dynamics of the system in non-equilibration conditions (Reuter et al., 2018).

### Brownian Dynamic Simulation

**FIGURE 2:**
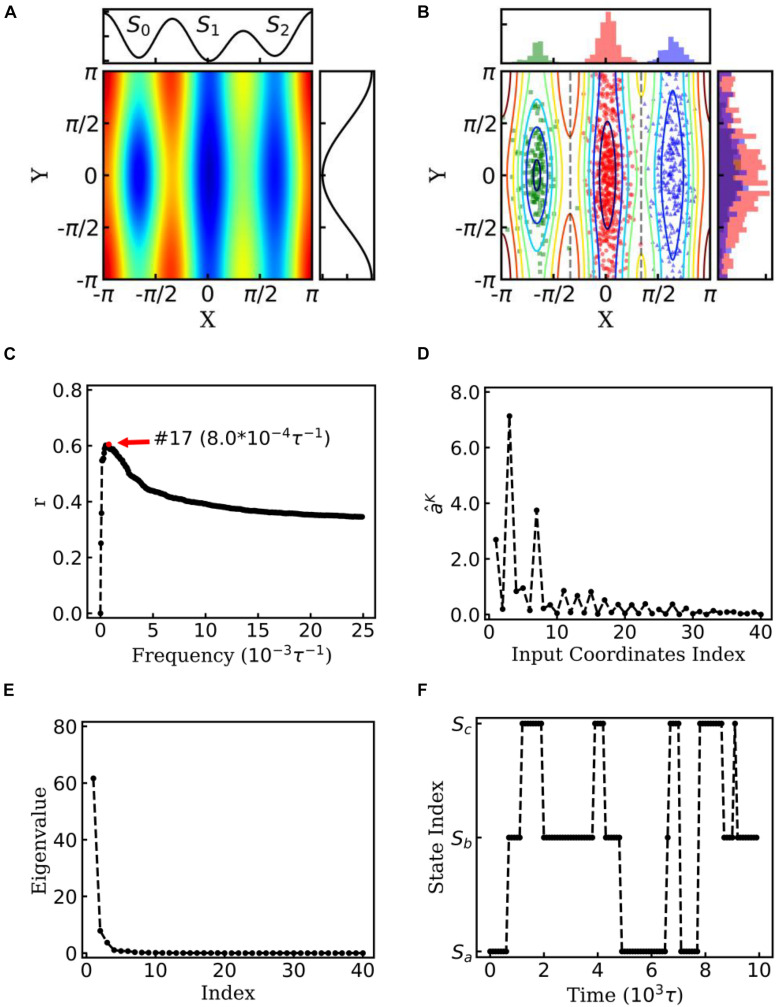
EspcTM on dynamic of Brownian particle. **(A)** The energetic landscape of the toy model. Here, the potential function of the landscape was $-\varepsilon \,\left\{\cos\left(x\right)+\sin\left(x\right)+2\,\cos\left(3\,x\right)+\frac{1}{2}\,\cos\left(y\right)+2\,\exp\left[-20\,{\left(x+\frac{2}{3}\,\pi \right)}^{2}-2\,y^{2}\right]\right\}$. Three potential wells from left to right were S_0_, S_1_, and S_2_. The well of S_1_ was deeper than that of the other two states, and the barrier between S_0_ and S_1_ was much higher than that between S_1_ and S_2_. The black line on the top and right panel represents the potential along line *y=0* and *x* = 0, respectively. **(B)** Red, green, and blue dots represent three states of the snapshots of trajectories. The histograms of each state were shown on the top and right panel in different colors. **(C)** Multiple correlation coefficients of ${\tilde{\varepsilon }}^{\text{K}}$ and all 40 conformational coordinates as a function of cutoff frequencies. Here, the maximum of the multiple correlation coefficient located at cutoff frequency equaling 8.0×10^−4^*τ*^−1^. **(D)** The regression coefficients for all 40 features. The coordinates corresponding to basis functions *sin*⁡(*x*),*cos*⁡(*x*),*andcos*⁡(2*x*) possessed large weights in the rescaling. **(E)** The eigenvalues in the PCA of trajectory-mapped vector. **(F)** A typical discretized trajectory.

For Brownian dynamic simulation, Brownian particles in the presence of a potential, *U*, are described by the Langevin equation

(7) $$m\,\frac{d\,v\,\left(t\right)}{d\,t}=-\nabla U\,\left(x\right)-\gamma \,v\,\left(t\right)+\xi \,\left(t\right)$$

where *ξ*(*t*) is a delta-correlated stationary Gaussian process with zero-mean. A two-dimensional Brownian particle was simulated on the surface with three potential wells in the toy model (see Figure 2A). Here, the potential *U*(*x*) was defined as:

(8) $$U\left(x\right)=-\varepsilon \{\cos\left(x\right)+\sin\left(x\right)+\frac{1}{2}\cos\left(y\right)+2\cos\left(3x\right)+2\exp\left[-20{\left(x+\frac{2}{3}\pi \right)}^{2}-2y^{2}\right]\}$$

with scaling parameter ε = 40. Multiple trajectories were generated from different initial sites randomly with extensive long simulations.

### MD Simulation

**FIGURE 3:**
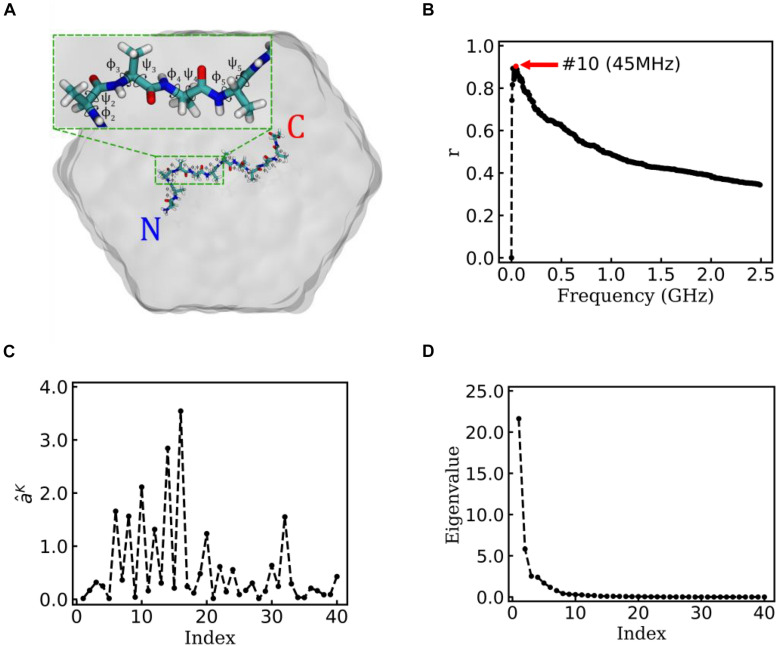
EspcTM on a typical trajectory of Ala_12_. **(A)** The typical conformation of Ala_12_ represented in sticks with labels of the 10 pairs of dihedral angles φ and ψ, solvated in SPC water molecules represented in gray surface. The inset figure shows the zoom-in of the segment contenting *φ*_*2∼5*_ and *ψ_2∼5_*. **(B)** Multiple correlation coefficients of ${\tilde{\varepsilon }}^{\text{K}}$ and all 40 conformational coordinates as a function of the cutoff frequency. The maximum of the multiple correlation coefficient located at a cutoff frequency equaling 45 MHz. **(C)** The regression coefficients for all 40 features. Most coefficients with large value correspond to the basis functions (sine and cosine) of *φ_2∼5_*. **(D)** The eigenvalues in the PCA of trajectory-mapped vector.

In the MD simulation, the termini of Ala_12_ were charged, which leads to versatile metastable structures (Noe et al., 2007). All atoms were modeled by using Amber03 force field. The molecule was solvated in a rhombic dodecahedral periodic box with the distance between the solutes and box boundary at least 10 Å. The SPC water model was used for solvation (see Figure 3A). The MD simulations were performed using the Gromacs package 4.6.5 (Hess et al., 2008). In the simulations, the covalent bonds involving H atoms were constrained by the LINCS algorithm, which allowed a time step of 2 fs. The long-range electrostatic interactions were treated with the particle-mesh Ewald method (Darden et al., 1993) with a grid spacing of 1.6 Å. The cutoff for the van der Waals interaction was set to 10 Å. The previous trajectory performed at high temperature was equilibrated by MD simulations for 100 ps at a constant pressure of 1 bar and a temperature of 500 K using Berendsen coupling (Berendsen et al., 1984). Then, the production simulations were performed in NVT ensemble at 500 K for 100 ns. All 50 systems extracted from high-temperature simulation had been iterated 100 ns in NVT ensemble at 300 K and recorded with time interval *τ* = 5ps. There are 20,000×50 frames in the analysis.

## Results and Discussion

The EspcTM method was first illustrated with a toy model, i.e., the dynamics of a Brownian particle on a two-dimensional surface. Then, it was applied to investigate the conformational dynamics of alanine dodecapeptide (Ala_12_), and a transition network between metastable states of Ala_12_ was constructed.

### Toy Model

In the toy model, a two-dimensional Brownian particle was moving in the field with three potential wells (see Figure 2A). Ten extensive long simulations, which started from different sites randomly, were performed to make the distribution of samples close to the theoretical values. Figure 2B shows the positions and distribution of the samples of these trajectories. In the analysis, *sin*⁡(*nθ*)*andcos*⁡(*nθ*) were selected as the basis functions. θ indicates the coordinate *xory*, and *n* = 1,…,10 for every coordinate in the EspcTM analysis of the toy model. Hence, the trajectories were mapped into a 40-dimensional functional space, e.g.,

(9) $$\begin{array}{c} \sin\left(x\right),\sin\left(y\right),\cos\left(x\right),\cos\left(y\right),\ldots \\ \sin\left(10\,x\right),\sin\left(10\,y\right),\cos\left(10\,x\right),\cos\left(10\,y\right) \end{array}$$

All values of the trajectories were normalized in every dimension before they were fitted with ${\tilde{\varepsilon }}^{K}$.

Figure 2C shows the multiple correlation coefficient between ${\tilde{\varepsilon }}^{\text{K}}$ and the values of these 40 features as a function of the cutoff frequency. There was a maximum multiple correlation coefficient at K = 17, and $\tilde{\varepsilon }={\tilde{\varepsilon }}^{17}-\epsilon^{17}$ was selected as the effective energy. Figure 2D shows regression coefficients between the energy ${\tilde{\varepsilon }}^{17}$ and features. As shown in Figure 2D, the basis functions *sin*⁡(*x*),*cos*⁡(*x*),and*cos*⁡(2*x*) possessed large weight in the rescaling. It should be noted that to consider the effect of the random force by solvation in Brown dynamics, additional energies with Gaussian distribution were added into the energies of the Brownian particle, so that information of potential was mixed with white noise in linear regression. PCA was performed on these effective energy rescaled samples. Figure 2E shows the eigenvalues in descending order. It is obvious that apart from the first two eigenvalues, other eigenvalues were very small. The first two eigenvectors were selected to compose the E-space of the toy model, as well as the mapping operator. By using the mapping operator *M*, composed by these two eigenvectors, all samples were mapped into the E-space.

By using the PCCA+ algorithm, all samples had been grouped into three states (shown by colored dots in Figure 2B). As shown in Figure 2B, these three states corresponded to the three wells in the potential. A discretized trajectory who visited all three states is shown in Figure 2F. The Markov transition matrix *P* was obtained based on the discretized trajectories (see Table 1). The stationary distribution, which corresponds to the distribution of the thermodynamic equilibrium, was obtained by the eigen-decomposition of the Markov transition matrix and shown in Table 1. As a benchmark, the distribution of equilibrium state predicted by the theory of statistical physics is shown in Table 1 as well. It is obvious that the result obtained by the EspcTM method is similar to the theoretical values. Furthermore, the Markov transition matrix contains kinetic information about the system as well. The lifetime of these states, which were calculated by the diagonal elements of the transition matrix, is also shown in Table 1. It was found that the state S_0_ possessed the lowest occurring probability but the longest lifetime. This indicated that the kinetically stable state was not the thermodynamically stable state for this dynamic system.

**TABLE 1:** Transition matrix and stationary distribution of the Markov model, distribution obtained by theory of equilibrium statistical physics, and lifetime of states for the dynamics of a Brownian particle.

	Transition matrix P			Stationary distribution	Theory	Lifetime (100)
	
	S0	S1	S2			
S0	0.882	0.069	0.049	0.184	0.186	8.5
S1	0.024	0.858	0.118	0.532	0.538	7.08
S2	0.032	0.221	0.747	0.284	0.276	3.99

### Dynamics of Alanine Dodecapeptide

Alanine dodecapeptide (Ala_12_), consisting of 12 alanine residues, is a typical model molecule for MD study (Noe et al., 2007). The MD trajectories of an Ala_12_ was used as an example to test the EspcTM method. According to the previous study (Gong and Zhou, 2010; Gong et al., 2015), sine and cosine of backbone dihedral angles (*φ*,*ψ*) were used as basis functions in the analysis of the MD trajectories of Ala_12_. Here, φ is defined as the backbone dihedral angle around the bond connecting C_*α*_ and N atoms and ψ is defined as the backbone dihedral angle around the bond connecting C_*α*_ and carbonyl carbon atoms (Hovmoller et al., 2002). There are 10 pairs of dihedral angles φ and ψ for Ala_12_ (see Figure 3A), and 40 basis functions were finally included in the analysis, e.g.,

(10) $$\sin\left(\psi_{i}\right),\sin\left(\varphi_{i}\right),\cos\left(\psi_{i}\right),\cos\left(\varphi_{i}\right)$$

Here, *i* = 1,…,10 indicates the index of dihedrals of Ala_12_ from N-terminal to C-terminal. Based on these basis functions, the EspcTM was first applied on a typical trajectory and then on all the 50 trajectories.

### State Transition of a Typical Trajectory

Figure 3B shows the result of the multiple linear regression between ${\tilde{\varepsilon }}^{\text{K}}$ and functions of the dihedral angles of Ala_12_ for a typical trajectory. There is a maximum of the multiple correlation coefficient, similar to the case of movement of Brownian particle, at 45 MHz (see Figure 3B). Therefore, the summary of the first 10 lowest frequencies of energy ${\tilde{\varepsilon }}^{10}$ was used in the analysis. The regression coefficients between the energy ${\tilde{\varepsilon }}^{10}$ and functions of dihedral angles are shown in Figure 3C. It was found that most factors with large weight corresponded to the basis function (sine and cosine) of *φ_2∼5_* (see the inset figure of Figure 3A). This indicates that the structure change near N-terminal contributes more to large-scale conformational change than C-terminal in this typical simulation trajectory.

**FIGURE 4:**
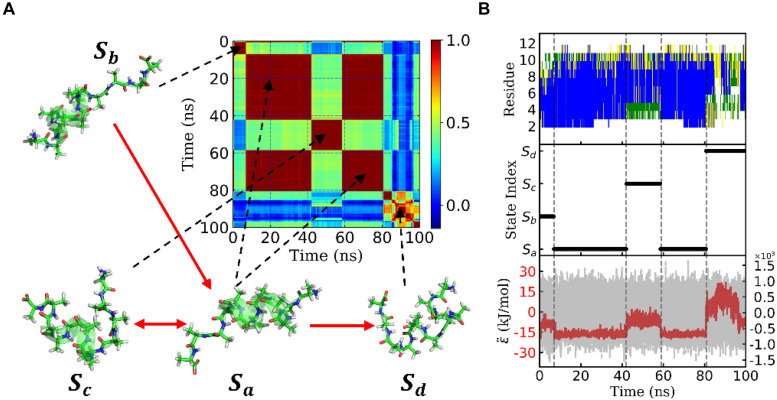
State transition of a typical trajectory of Ala_12_. **(A)** Similarity matrix and typical conformations in the metastable states and their transitions. The color indicated the degree of similarity. Red means high similarity. The transitions were implied from the transition probability matrix. **(B)** Secondary structure analysis of the typical trajectory by DSSP was shown in the upper panel. The blue, green, yellow, and white patterns represented α-helix, bend, turn, and coil, respectively. The discretized trajectory was shown in the middle panel. The states corresponded to the similarity matrix in panel **(A)**. In the lower panel, the effective energy for this typical trajectory was exhibited in the red dashed curve and the original potential energy was in the gray curve as background. Both curves shared the same *x*-axis but with *y*-axis in different scales. The effective energy’s *y*-axis was on the left with an amplitude of about 20 kJ/mol, while the original potential energy’s *y*-axis was on the right with an amplitude of about 1.2×10^3^ kJ/mol. Here, both effective energy and original potential energy had been zero-centered.

Figure 3D shows the eigenvalues of weighted samples of this trajectory. As shown in Figure 3D, the following analysis on this trajectory was performed in the space made up of the first six eigenvectors. Figure 4A shows the similarity matrix and the representative structure of the trajectory. It was obvious that there were four metastable states in the trajectory. The discretized trajectory is shown in the middle panel of Figure 4B. The secondary structure of the peptide was analyzed by DSSP (Kabsch and Sander, 1983; Touw et al., 2015) and shown in the top panel of Figure 4B. The simulation started from a structure with some of the N-terminal α-helix formed (also see the representative structure), i.e., the state S_b_. This state was unstable and only existed about 6.4 ns in the 100-ns trajectory. The α-helix formed in this state acted as a nucleus that promoted the formation of the α-helix of the C-terminal of the Ala_12_. Then, the trajectory transited to the S_a_ state, in which most of the residues of the peptide formed the α-helix structure. State S_a_ was more stable than state S_b_. It appeared two times in this trajectory and existed about 58.0 ns in total. However, between the two occasions of the state S_a_, the α-helix of two termini had been temporally uncoiled and interacted with the α-helix in the middle of the peptide, i.e., the state S_c_. This state is unstable and existed only for 16.4 ns in this trajectory. After the state S_c_, the peptide folded to the state S_a_ again. Finally, the peptide unfolded into a random coil, i.e., state S_d_, with low structural similarity.

The bottom panel of Figure 4B shows effective energy as a function of time for this trajectory. It was calculated from the total energy of the whole biosystem, including the peptide and water molecules. Initially, the energy caused by the conformational change of the peptide was concealed by the noise of the dynamics of water molecules as well as the fluctuation of itself. It seemed that the total energy (shown in gray) varied randomly and dramatically. However, by using the FFT and regression, we obtained the effective energy (shown in red). It was synchronous with conformational change and state transition of the peptide. More interestingly, the effective energy of stable state, state S_a_, was much lower than the other three states, in which most of the α-helix was formed. This implied that the stability of this state was supported by energy. On the other hand, the state S_d_ possessed the highest energy and large conformational variations. This implied that the unfolded coil structure was stabled by the entropy.

### Transition Network of Ala_12_

**FIGURE 5:**
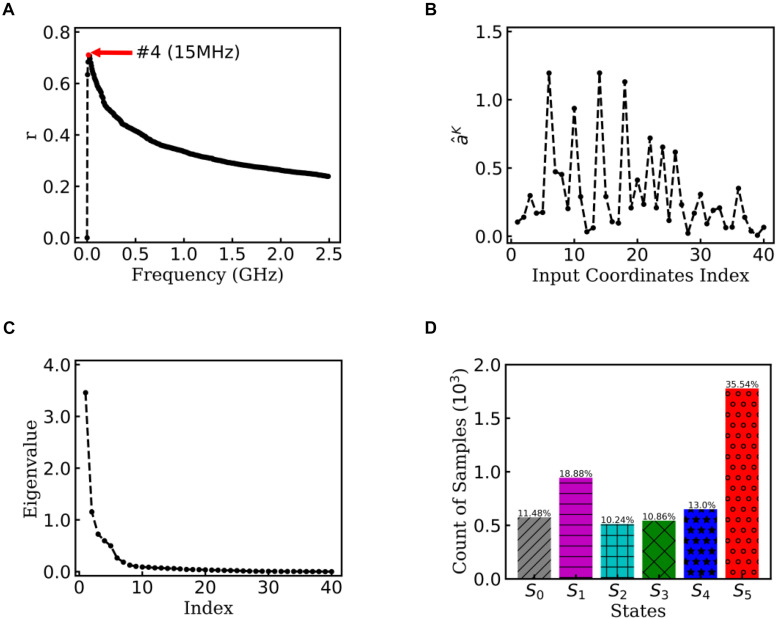
EspcTM on 50 trajectories of Ala_12_. **(A)** Multiple correlation coefficients of regression between ${\tilde{\varepsilon }}^{\text{K}}$ and features as a function of cutoff frequencies. The maximum was at 15 MHz. **(B)** The regression coefficients for all 40 features. **(C)** The eigenvalues in the PCA of trajectory-mapped vector. **(D)** The observed probability for each state in all 50 trajectories.

To obtain statistically significant conclusions, we performed the analysis of EspcTM method on 50 MD trajectories. Figure 5A shows the result of the multiple linear regression between ${\tilde{\varepsilon }}^{\text{K}}$ and functions of the dihedral angles of Ala_12_ for these 50 trajectories. The maximum of the multiple correlation coefficient was found at the frequency equal to 15 MHz. The summation of the first four lowest frequencies of energy ${\tilde{\varepsilon }}^{4}$ was used in the analysis. Figure 5B shows the regression coefficients between the energy ${\tilde{\varepsilon }}^{4}$ and features. It consistently showed that *φ_2∼5_* played important roles in the dynamics of the Ala_12_ though there was a phase shift on *φ_2∼5_* caused small weights on the cosine of *φ_2∼5_*. This indicates that local structure changes near the N-terminal, especially the *φ_2∼5_*, were the major contributors to the slow conformational change of the Ala_12_. According to the result of the PCA on the weighted feature space, the clustering algorithm was performed in the space made up of the first 10 eigenvectors, whose sum was over 90% sum of variation (see Figure 5C). Every trajectory was divided into 100 pieces. Thus, there were 5,000 vectors, which represent 100×50 trajectory pieces. Six states were identified from these 50 trajectories.

**FIGURE 6:**
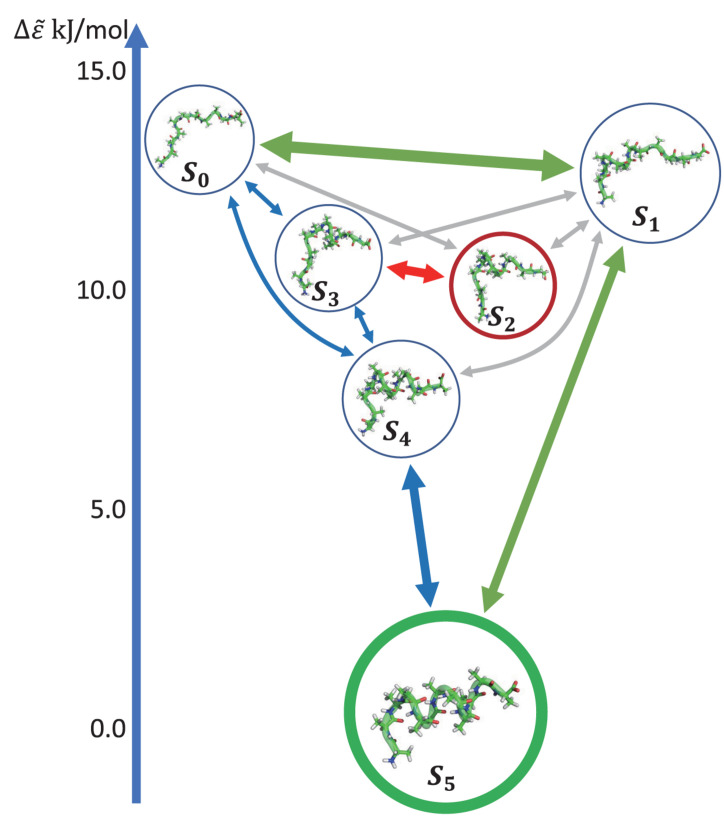
Dynamics network of Ala_12_. The metastable states were shown by the cartoon structures of their typical conformations and rearranged by their average effective energy in vertical. The size of circles around the pictures indicated the occurring probability in the stationary mode. The arrows showed the main transitions between six states in the equilibrium state. The pathway was indicated by the color of the arrows. The transitions were shown in three levels according to the transition frequency, i.e., ∼30, ∼10, and ∼5μs^–1^, and indicated by the linewidth of the arrows.

Figure 5D shows the histogram of these six states. Here, the state transitions were obtained from the 50 trajectories with the lag time 1.0 ns. The transition matrix and stationary distribution are shown in Table 2. It was found that the stationary distribution obtained by the transition matrix was consistent with the histogram. The state S_5_ had a much higher occurring probability than that of other states in the equilibrium state. Figure 6 displays these six states, represented by their typical structures in cartoons, along with their average effective energy in vertical. The unfolded states S_0_, in which peptide unfolded into a random coil, possessed the highest energy and located at the top of the figure. The folded state S_5_, in which the peptide folded into α-helices, possessed the lowest effective energy and located at the bottom of the figure. Between these two states, the peptide was half-folded. In the state S_1_, a helix was formed in the N-terminal of the peptide. In states S_2_, S_3_, and S_4_, some helices were formed in the C-terminal. A remarkable gap between the effective energy of state S_4_ and state S_5_ separated the folded state from the other five states. This implied that the energy is the reason for the stability of the folded state.

**TABLE 2:** Transition matrix and stationary distribution of the Markov model and lifetime of states for the dynamics of Ala_12_.

	Transition matrix						Stationary distribution	Lifetime (ns)
	
	S0	S1	S2	S3	S4	S5		
S0	0.610	0.276	0.038	0.031	0.036	0.009	0.114	2.61
S1	0.170	0.693	0.021	0.037	0.026	0.053	0.187	3.30
S2	0.042	0.037	0.758	0.115	0.030	0.018	0.103	4.16
S3	0.032	0.064	0.109	0.725	0.050	0.020	0.109	3.67
S4	0.031	0.038	0.024	0.042	0.798	0.067	0.131	4.97
S5	0.003	0.027	0.005	0.006	0.025	0.934	0.356	15.15

Furthermore, we obtain the dynamics and kinetics of the system based on the transition matrix. Figure 6 shows the main transition between six states in lines with arrows. The most frequent transition, about 32 μ*s*^−1^, occurred between the state S_0_ and S_1_ due to the high flexibility of the peptide in these two states. This high transition frequency made the lifetime of these two states lower than that of states S_2_, S_3_, and S_4_, though the occurring probabilities of these two states were a little higher than the other three states. In the transition network, there were two main folding pathways from the unfolded state to the folded state. The fast folding pathway, which passed through state S_1_ and was shown by green arrows, formed the α-helices from the N-terminal to the C-terminal directly. The slow folding pathway, which involved states S_2_, S_3_, and S_4_, was shown by blue and red arrows and was more complex than the fast one. In this pathway, the α-helices formed from the C-terminal to the N-terminal, i.e., passed through states S_3_ and S_4_ sequentially. The misfolded state S_2_ connected with state S_3_. A detailed structural study showed that the structures of states S_2_ and S_4_ were very similar. However, some misfolded residues hindered the formation of the N-terminal helix in the state S_2_. To reach the folded state, it must unfold into state S_3_. These results indicated that the N-terminal helix plays a vital role in the folding of the peptide in kinetics. It is consistent with the aforementioned result of linear regression, that the *φ_2∼5_* of the peptide possessed large rescaling factors, as well as the results by other experimental groups, that alanine-rich peptides folded into the α-helix in the N-terminal at first (Millhauser et al., 1997; Yoder et al., 1997). It must be noted that, as we mentioned before, the biomolecules are intrinsically dynamic (Chodera et al., 2007) and the unfolded states of the peptide were transferred to each other frequently. These two pathways only described the major folding process of Ala_12_. Some minor branches in the folding pathways also existed.

## Conclusion

In this work, we introduced our EspcTM method by applying it to investigate the movement of Brownian particle and conformational dynamics of Ala_12_ in this work. In the study of Brownian particle, by using the EspcTM method, we obtained three states from simulation trajectories. The regions of the states given by EspcTM are in accordance with the potential wells of the landscape. In addition, the equilibrium distribution obtained by the kinetic transition network-based Markov chain theory was consistent with the theoretical result. In the study of Ala_12_, a meaningful kinetic transition network was obtained to describe the folding behavior of Ala_12_. The effective energy, which was filtered from the total potential energy of simulation trajectories by FFT and multiple linear regression, was shown to be an efficacious order parameter to describe the conformational change of Ala_12_. We showed that the folding process of Ala_12_ was synchronous with the change of effective energy. The folded state, in which most of the residues were in helices, possessed the lowest effective energy and was most stable in thermodynamics. Two major folding pathways were also found in the kinetic network. The N-terminal helix of the Ala_12_ was found to play an important role in the folding of Ala_12_ in both thermodynamics and kinetics. This is consistent with previous experimental result. Thus, the EspcTM is expected to be a powerful tool for studies of dynamics of complex systems and should be applied to studies of dynamics of large biomolecule systems to improve our understanding of the thermodynamics and kinetics of biomolecular systems.

Technically, the EspcTM method is an analysis framework based on the TM method. It identifies metastable states from simulation data and constructs the transition network between the states based on the theory of Markov chain. Different from the TM method, we provided a *de novo* solution to obtain an analysis space, named as E-space, to describe the slow processes in the EspcTM method. This solution is based on a parameter-free optimization approach. Thus, the EspcTM method is friendly to inexperienced users. The E-space is independent from the TM method. It is convenient to use it in the MSM method. For the experienced users, especially those with knowledge on the dynamics of system, they can set cutoff frequency manually as well. Furthermore, as an extension of the EspcTM method, some new transfer functions, such as logistic function and ReLU, can also be used in the energy filter process. The wavelet analysis method can be used in transforming the energy between time domain and frequency domain.

## Data Availability Statement

The original contributions presented in the study are included in the article/supplementary material, further inquiries can be directed to the corresponding author.

## Author Contributions

GZ and ZW designed the study. ZW collected data and carried out the calculation. ZW, XZ, and GZ wrote the manuscript. All authors contributed to the article and approved the submitted version.

## Conflict of Interest

The authors declare that the research was conducted in the absence of any commercial or financial relationships that could be construed as a potential conflict of interest.
